# A study of the Interaction Between Cetirizine and Plasma Membrane of Eosinophils, Neutrophils, Platelets and Lymphocytes using A fluorescence Technique

**DOI:** 10.1155/S0962935194000335

**Published:** 1994

**Authors:** A. Kantar, N. Oggiano, P. L. Giorgi, J-P. Rihoux

**Affiliations:** 1 Pediatric Clinic University of Ancona Italy; 2 UCB Pharma Medical Department Braine I’Alleud Belgium

## Abstract

The effect of cetirizine on plasma membrane fluidity and heterogeneity of human eosinophils, neutrophils, platelets and lymphocytes was investigated using a fluorescence technique. Membrane fluidity and heterogeneity were studied by measuring the steady-state fluorescence anisotropy and fluorescence decay of 1-(4- trimethylammonium-phenyl)-6-phenyl-1, 3, 5-hexatriene (TMA-DPH) incorporated in the membrane. The results demonstrate that cetirizine (1 μg/ml) induced a significant increase in the Hpid order in the exterior part of the membrane and a decrease in membrane heterogeneity in eosinophils, neutrophils and platelets. Moreover, cetirizine blocked the PAF induced changes in membrane fluidity in these cells. Cetirizine did not influence significantly the plasma membrane of lymphocytes. These data may partially explain the effect ofcetirizine on inflammatory cell activities.


Mediators of Inflammation 3, 229–234 (1994)

### A study of the interaction between cetirizine and plasma membrane of eosinophils, neutrophils, platelets and lymphocytes using a fluorescence technique

A. Kantar,[1,CA] N. Oggiano,[1] P. L. Giorgi[1] and J-P. Rihoux[2]

[1]Pediatric Clinic, University of Ancona, Italy; [2]UCB Pharma, Medical Department, Braine l'Alleud, Belgium

[CA] Corresponding Author

**THE effect of cetirizine on plasma membrane fluidity and heterogeneity of human eosinophils, neutrophils, platelets and lymphocytes was investigated using a fluorescence technique. Membrane fluidity and heterogeneity were studied by measuring the steady-state fluorescence anisotropy and fluorescence decay of 1-(4-trimethylammonium-phenyl)-6-phenyl-1, 3, 5-hexatriene (TMA–DPH) incorporated in the membrane. The results demonstrate that cetirizine (1 μg/ml) induced a significant increase in the lipid order in the exterior part of the membrane and a decrease in membrane heterogeneity in eosinophils, neutrophils and platelets. Moreover, cetirizine blocked the PAF induced changes in membrane fluidity in these cells. Cetirizine did not influence significantly the plasma membrane of lymphocytes. These data may partially explain the effect of cetirizine on inflammatory cell activities.**

**Key words:** Cetirizine, Eosinophil, Fluorescence, Lymphocyte, Neutrophil, Plasma membrane, Platelet, TMA–DPH

### Introduction

Cetirizine, a piperazine derivative and carboxylated metabolite of hydroxyzine in humans, is a long-acting non-sedating, histamine $H_1$-receptor antagonist.[1] Apart from its potency in inhibiting histamine-induced reactions, several antiallergic effects of cetirizine are not explained by antagonism of the $H_1$-receptor. Recent investigations have demonstrated for instance that cetirizine has a modulating effect on different cells possibly involved in the allergic inflammatory phenomenon. Among these are eosinophils (Eos), neutrophils (PMN) and platelets (Plt).[1] Cetirizine inhibits Eos chemotaxis,[2–4] accumulation,[5–8] adherence and activation.[9,10] Moreover, it inhibits an antigen-induced PMN accumulation in atopic skin.[9,10] In addition, oxygen free radical generation following IgE dependent activation of Plt and antigen-induced platelet-dependent leukopenia are inhibited by cetirizine.[11,12] All of these cellular activities are the result of complex events mediated by plasma membrane.

It has long been recognized that cell membranes have fundamental physiological tasks, in addition to their action as selective boundaries. In fact, most of the cellular biochemical and biophysical events occur in the membrane, where strict structural and dynamic features provide the control mechanisms.[13]

Membrane fluidity has an important role in modulating cell functions, affecting the conformation of membrane proteins and the exposure and diffusion of membrane components. Fluidity is a complex physico-chemical feature that depends upon mobility and order of membrane constituents.[13,14] This study was performed to discern the effect of cetirizine on membrane fluidity and heterogeneity of human Eos, PMN, Plt and lymphocyte using a fluorescence technique. As a fluorescent probe we employed 1-(4-trimethylammonium-phenyl)-6-phenyl-1, 3, 5-hexatriene (TMA–DPH).

### Material and Methods

Blood samples were obtained from healthy donors. All subjects were without signs of acute infection and had not suffered from infection in the previous 3 weeks. None of the subjects enrolled in the study was undergoing anti-inflammatory or antihistamine therapy.

For the preparation of eosinophils, blood samples were obtained from ten subjects having a documented seasonal allergic rhinitis and a positive skin test for only grass pollen. Samples were obtained before the grass pollen season. Patients were not under specific hyposensitization and had not taken any medications in the previous 2 months.

*Preparation of PMN:* PMN were isolated from freshly drawn blood using a Mono-Poly Resolving Medium (ICN Biomedicals, Milan, Italy) as described previously.[15] A sample of 3.5 ml of heparinized blood was layered onto 3 ml of Mono-Poly Resolving Medium and centrifuged at 300 × **g** for 45 min at room temperature. The PMN band was transferred to a sterile tube. Cells were washed and resuspended

© 1994 Rapid Communications of Oxford Ltd in Krebs–Ringer phosphate solution (KRP) supplemented with 5 mM glucose. The total PMN count was obtained using a Sysmex E-2500 whole blood analyser.

*Preparation of eosinophils:* Eosinophils were isolated according to Koenderman *et al.*[16] Polymorphonuclear leukocytes were isolated as described previously[15] and cells were suspended at a final concentration of $75 \times 10^6$ cells/ml. Cells were incubated with fMLP (final concentration 10 nM) for 10 min. One ml of the cell suspension was layered on a discontinuous double-layered Percoll (Pharmacia, Uppsala, Sweden) gradient of densities 1.082 and 1.100 g/ml and centrifuged for 15 min at 1 000 × **g**. Eos were collected from the interface between the two Percoll layers and washed with KRP and glucose. The differential cell count, determined from May–Gruenwald/Giemsa stained smear, indicated that isolates were 90% Eos.

*Preparation of platelets:* Ten ml of freshly drawn acid–citrate–dextrose anticoagulated blood was centrifuged at 120 × **g** for 15 min at 37°C to prepare platelet rich plasma. Plt were isolated and washed according to Kubina *et al.*[17] Plt were finally resuspended in Tyrode's buffer (137 mM NaCl, 2 mM KCl, 12 mM $NaHCO_3$, 0.3 mM $NaH_2PO_4$, 1 mM $MgCl_2$, 5 mM glucose) with 0.1% bovine serum albumin and Apyrase (Sigma Chemical Co., St Louis, MO). The Plt suspension was adjusted to a concentration of $4 \times 10^8$ Plt/ml.

Phase-contrast microscopy (Diaplan, Leica, Milan, Italy) showed that, under the conditions used, control and TMA–DPH Plt remained discoid and functional, being sensitive to thrombin (1 U/ml).[18]

*Preparation of lymphocytes:* Lymphocytes were prepared from freshly drawn blood using Lymphocyte Separation Medium LymphoSep (ICN Biomedicals) as described previously.[19] Two ml heparinized blood diluted 1:1 with KRP solution was layered over 4 ml of LymphoSep and centrifuged at 400 × **g** for 20 min at room temperature. The lymphocytes band was washed and resuspended in KRP solution.

Cell count was determined using a Sysmex E-2500 whole blood analyser. Cells were immediately used for fluorescence measurements. Labelling with TMA–DPH was carried out in the dark. TMA–DPH was added to the cell suspension to give a final concentration of 1 μM. Fluorescence measurements were performed before and after addition of cetirizine (1 μg/ml). PAF was later added to the samples at a final concentration of $10^{-7}$ M.

*Fluorescence studies:* Steady-state fluorescence anisotropy ($r_s$) measurements were performed at 37°C with a Perkin–Elmer Spectrofluorometer MPF-66, equipped with a Perkin–Elmer 7300 Personal Computer for data acquisition and elaboration as described previously.[20] The computer program calculated fluorescence anisotropy by using the expression $(I_{\parallel} - I_{\perp} \times g)/(I_{\parallel} + [2I_{\perp} \times g])$ where $g$ is an instrumental correction factor, $I_{\parallel}$ and $I_{\perp}$ are respectively the emission intensities with polarizers parallel and perpendicular to the direction of the polarized exciting light.

Fluorescence lifetime measurements were performed as described previously[21] with a multifrequency phase fluorometer. The instrument was equipped with an ADC interface (ISS Inc., Champaign, IL, USA) for data collection and analysis; the excitation wavelength was set at 325 nm (ultraviolet line of a helium/cadmium laser, Liconix Model 4240 NB). The range of modulation frequencies used for TMA–DPH was 17–130 MHz. Data were accumulated at each modulation frequency until the standard deviation of the phase and modulation values were below 0.1° and 0.002, respectively. The fluorescence was measured through a long-pass filter (type RG 370 from Janos Tecnology, Townshend, VT, USA) which showed negligible luminescence. The experimental data were analysed by a model that assumes a continuous distribution of lifetime values characterized by Lorentzian shape centred at a time C and having a width W.[22] For this analysis the program minimizes the reduced chi-squared defined by an equation reported by Fiorini *et al.*[23] The temperature of the samples was maintained at 37°C with an external bath circulator.

*Analysis of the results:* The data were compared by Student's *t*-test. The level of significance was taken at $p < 0.05$.

### Results

The background phospholipid fluorescence of cells (Eos, PMN, Plt or lymphocytes) was checked prior to each measurement and was less than 0.5% of the fluorescence when TMA–DPH was added. The fluorescence intensity of cetirizine in the suspension solution at the used concentrations was negligible and did not increase upon the addition of TMA–DPH or PAF.

In the control cell samples (without cetirizine) a stable $r_s$ value was maintained for the following 30 min. The results of $r_s$ values in Eos, PMN and Plt before and after addition of cetirizine, 1 μg/ml, and PAF are shown in Figs 1–3. When cetirizine was added at a concentration of 0.1 μg/ml no significant changes of $r_s$ values were observed (data not shown). The effect of PAF on these cells was not blocked in the presence of cetirizine. However, at a concentration of 1 μg/ml, cetirizine induced a significant and stable increase in $r_s$ value. The subsequent addition

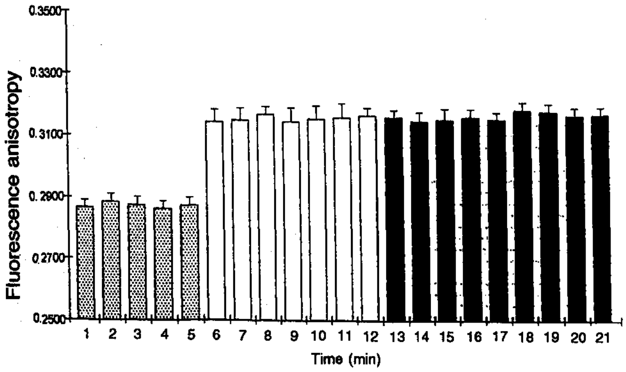

FIG. 1. Steady-state fluorescence anisotropy ($r_s$) of TMA–DPH at 37°C in Eos plasma membranes before (▩) and after (□) addition of cetirizine and PAF (■). Values are expressed as the mean + 2S.D. of ten samples. The addition of cetirizine induced a significant increase in $r_s$ value ($p < 0.0001$).

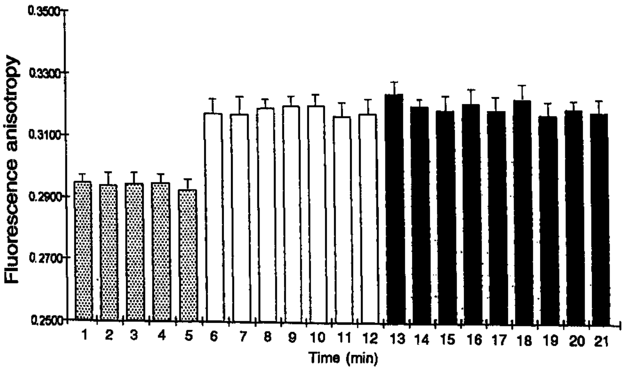

FIG. 2. Steady-state fluorescence anisotropy ($r_s$) of TMA–DPH at 37°C in PMN plasma membranes before (▩) and after (□) addition of cetirizine and PAF (■). Values are expressed as the mean + 2S.D. of ten samples. The addition of cetirizine induced a significant increase in $r_s$ value ($p < 0.0001$).

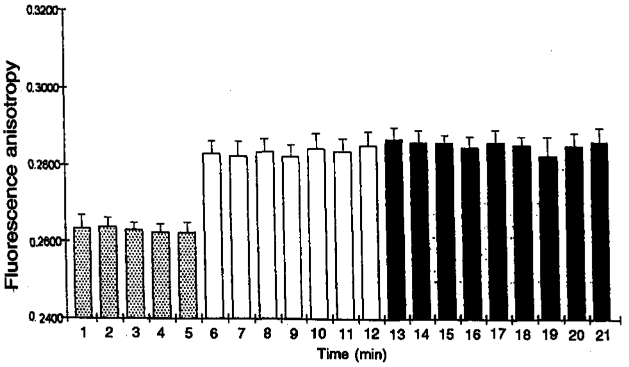

FIG. 3. Steady-state fluorescence anisotropy ($r_s$) of TMA–DPH at 37°C in Plt plasma membranes before (▩) and after (□) addition of cetirizine and PAF (■). Values are expressed as the mean + 2S.D. of ten samples. The addition of cetirizine induced a significant increase in $r_s$ value ($p < 0.0001$).

of PAF to cells did not induce significant changes in membrane fluidity ($p > 0.5$). The addition of cetirizine to lymphocytes neither induced significant changes in $r_s$ values, nor reduced the effect of PAF addition (Fig. 4).

Figures 5–7 show the results of the lifetime distribution of analysis of TMA–DPH decay in cell membranes in the absence (A) or presence (B) of cetirizine (1 μg/ml). Eos in the absence of cetirizine

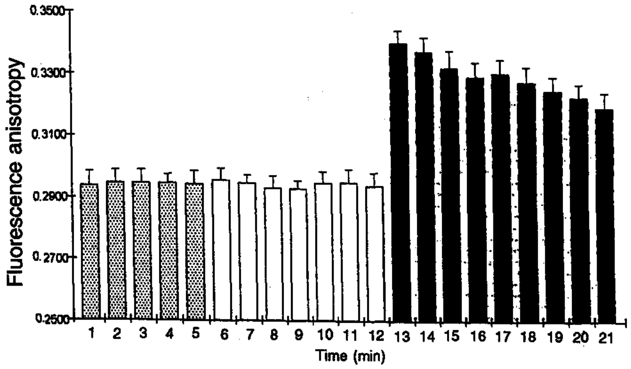

FIG. 4. Steady-state fluorescence anisotropy ($r_s$) of TMA–DPH at 37°C in lymphocyte plasma membranes before (▩) and after (□) addition of cetirizine and PAF (■). Values are expressed as the mean + 2S.D. of ten samples. The addition of PAF induced a significant increase in $r_s$ value ($p < 0.0001$).

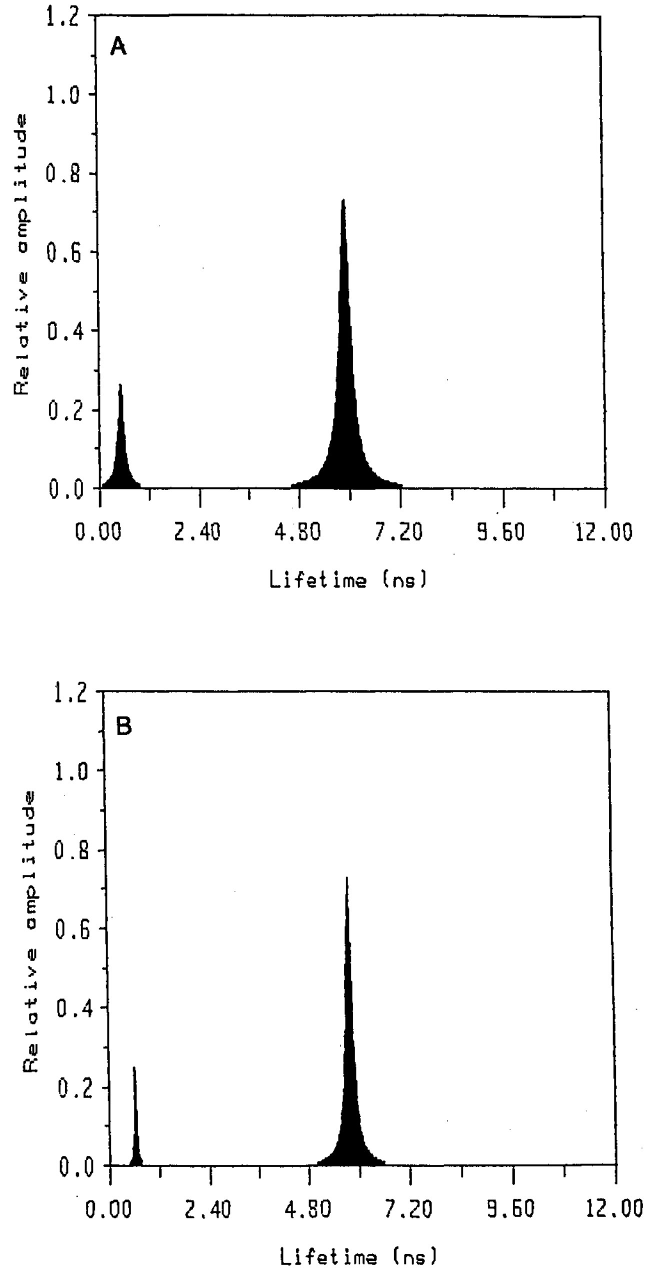

FIG. 5. (A) TMA–DPH lifetime distribution in Eos plasma membrane. (B) TMA–DPH lifetime distribution in Eos plasma membrane after addition of cetirizine (1 μg/ml).

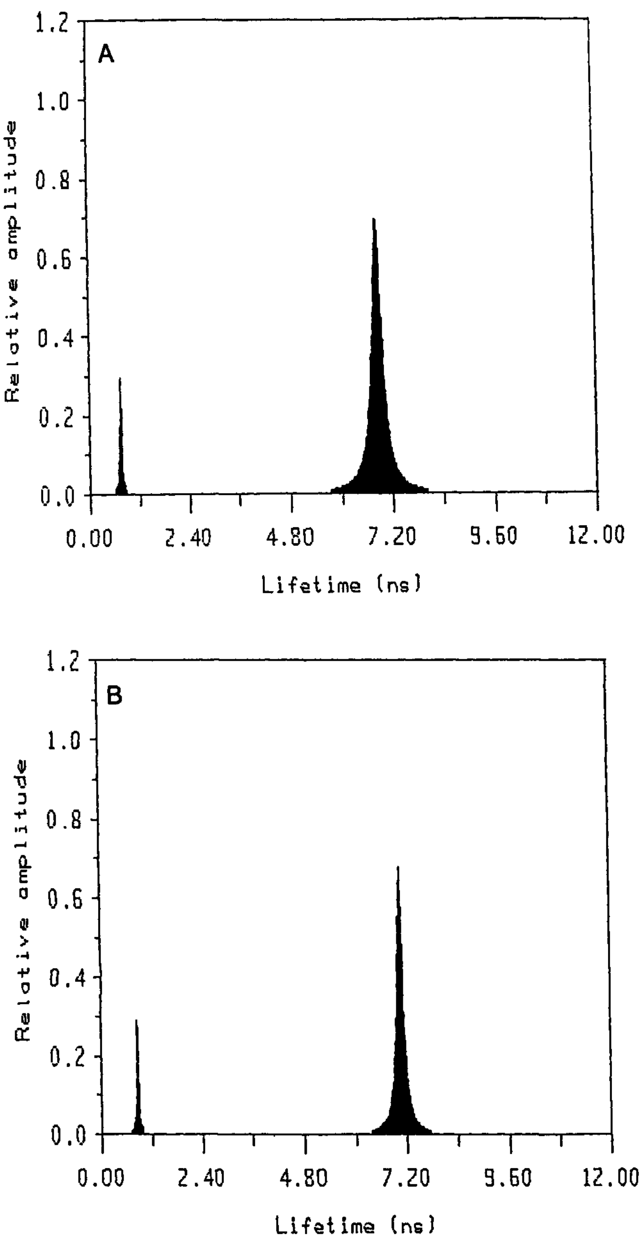

FIG. 6. (A) TMA–DPH lifetime distribution in PMN plasma membrane. (B) TMA–DPH lifetime distribution in PMN plasma membrane after addition of cetirizine (1 μg/ml).

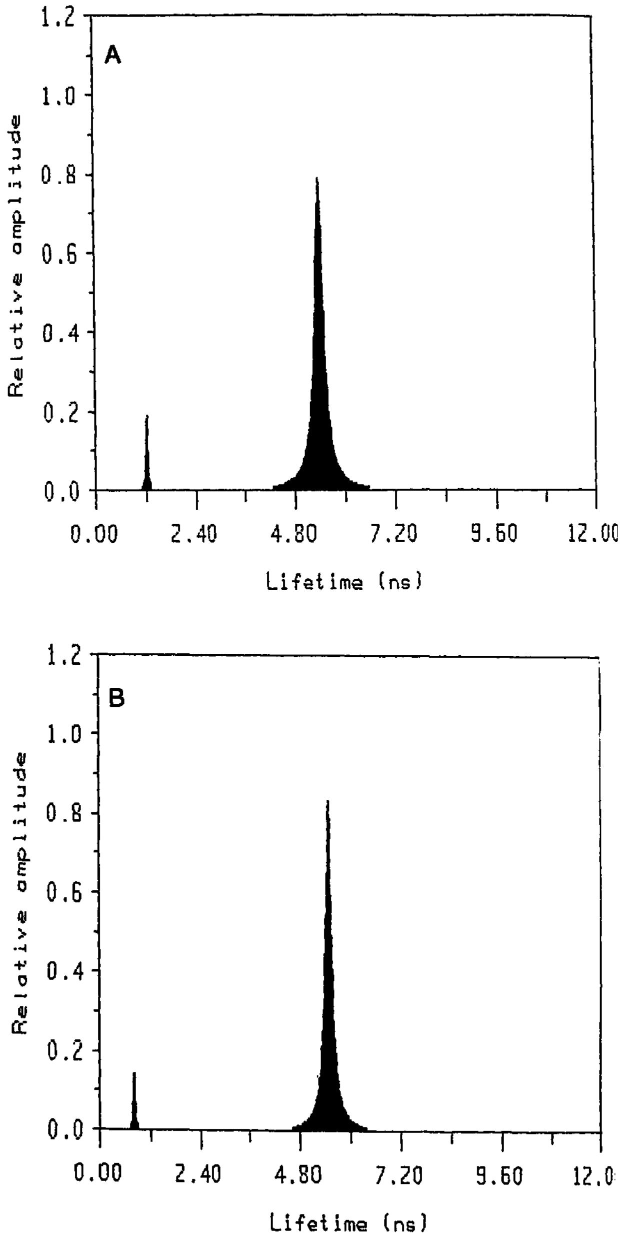

FIG. 7. (A) TMA–DPH lifetime distribution in Plt plasma membrane. (B) TMA–DPH lifetime distribution in Plt plasma membrane after addition of cetirizine (1 μg/ml).

(Fig. 5A) showed a two-component distribution; a long component with an average lifetime value ($C_L$) of 5.919 ns and fractional intensity ($f_L$) of 0.734, and a short component with an average lifetime value ($C_S$) of 0.512 ns and fractional intensity ($f_S$) of 0.266. The distribution width of the long component ($W_L$) was 0.307 ns and 0.178 for the short component ($W_S$). After the addition of cetirizine to Eos a significant reduction in $W_L$ was observed (value after addition of cetirizine 0.187 ns), whereas $C_L$, $f_L$, $C_S$, $W_S$ and $f_S$ did not change significantly (Fig. 5B).

PMN in the absence of cetirizine showed a two component distribution, the long component was characterized by a $C_L$ of 6.879 ns, $W_L$ of 0.208, and $f_L$ of 0.696, and the short component $C_S$ of 0.743 ns, $W_S$ of 0.050 and $f_S$ of 0.304 (Fig. 6A). After the addition of cetirizine a significant reduction in $W_L$ value was observed (0.167). $C_L$, $f_L$, $C_S$, $W_S$ and $f_S$ were not significantly changed after the addition of cetirizine (Fig. 6B).

Plt in the absence of cetirizine showed a long component with a $C_L$ of 5.402 ns, $W_L$ of 0.254 and $f_L$ of 0.790, and a short component with a $C_S$ of 1.210 ns, $W_S$ of 0.050 and $f_S$ of 0.210 (Fig. 7A). The addition of cetirizine reduced the $W_L$ significantly (0.192 *vs.* 0.254). However, $C_L$, $f_{SL}$, $C_S$, $W_s$ and $f_S$ were not significantly changed (Fig. 7B).

Lymphocytes in the absence of cetirizine showed a long component with a $C_L$ of 5.418 ns, $W_L$ of 0.201 and $f_L$ of 0.831, and a short component with a $C_S$ of

0.522 ns, $W_S$ of 0.050 an $f_S$ of 0.169. The addition of cetirizine did not induce significant changes in these parameters.

### Discussion

The plasma membrane, located at the interface between the external environment and the cell metabolic machinery, is the site of the regulatory events which control functional activities and cellular processes. Communication and stimulus–response coupling across and along the cell membrane provide a basis for functions such as motility, adhesion, aggregation and immune response.[24]

To mediate such functions, lipids act as barriers, solvents, anchors, activators and conformational stabilizers for proteins that carry out specific functions.[25] The membrane functional diversity is reflected in the wide variety of lipids and proteins that compose different membranes.

The fluid mosaic model describes the cell membrane as a fluid two-dimensional lipid bilayer matrix with its associated proteins that allows for lateral diffusion of both lipids and proteins in the plane of the membrane.[26] Specific lipid–lipid and lipid–protein interactions result in a precisely controlled yet highly dynamic architecture of the membrane components, as well as in its selective modulation by the cell and the environment.[27] Different modes of organization of the compositionally and functionally differentiated domains would correspond to different functional states of the membrane.[28] A key issue in the fluid mosaic model is membrane fluidity which reflects the physical properties of lipids and has been found to correlate with the activity of several membranes.[29] A variety of spectroscopic techniques have been employed for the investigation of the structure and dynamic properties of synthetic and natural membranes. In particular, fluorescence techniques have been used to investigate membrane fluidity by means of extrinsic probes embedded in the membrane. 1,6-diphenyl-1,3,5-hexatriene (DPH) and its derivatives are perhaps the most commonly used probes to investigate the physical structural properties of membranes at the molecular level.[30–32] TMA–DPH is a fluorescent hydrophobic probe, introduced in 1981 by Prendergast *et al.*[33] In addition to its favourable photophysical characteristics (similar to those of DPH) in membranes, it remains anchored by its charged trimethylammonium group at the level of the lipid–water interface region for at least 30 min allowing measurements in intact cells.[31]

TMA–DPH $r_s$ values reflect the packing of membrane lipid fatty acid chains and can be related to the order parameter, S, if certain precautions are taken.[34,35] Lipid fluidity may be defined as the reciprocal of the lipid structural order parameter S[34] and thus an increase of TMA–DPH $r_s$ value corresponds to a decrease in membrane fluidity. In this study it was observed that cetirizine at a concentration of 1 μg/ml increased significantly TMA–DPH $r_s$ values in Eos, PMN and Plt. This indicates that cetirizine (1 μg/ml) induces a decrease in fluidity which reflects an increase in lipid ordering in the exterior part of the plasma membrane of cells. A lower concentration of cetirizine, 0.1 μg/ml, did not induce significant changes in $r_s$ values (data not shown). In lymphocytes cetirizine did not influence TMA–DPH $r_s$ values and this confirms previous data demonstrating that this drug does not affect lymphocyte function *in vitro*.[36] To discern if these changes in membrane fluidity induced by cetirizine could influence the previously observed effect of PAF on these cells,[18,37,38] PAF $10^{-7}$ M was added to these samples. In Eos, PMN and Plt the effect of PAF was abolished by cetirizine.

TMA–DPH fluorescence decay depends on the dielectric constant of the medium in which the probe is embedded.[39] Therefore the width of the lifetime distribution can be related to the different physicochemical properties of the environment surrounding the probe. The distribution analysis, although based on phenomenological grounds, offers a good description of membrane heterogeneity.[40,41] In our distribution analysis it was necessary to include a second component at shorter lifetime with a low fractional intensity. The origin of this component is still debated; for DPH this component has been referred to as a photochemical derivative of the probe[41] or alternatively, it can represent a fraction of the probe localized in a very polar environment.[42] In this study the short component was not influenced by the addition of cetirizine. The data for TMA–DPH lifetime distribution show that cetirizine induced a decrease in the long component width in Eos, PMN and Plt. No effect was observed on lymphocyte membrane. These results indicate that cetirizine induces a decrease in membrane heterogeneity of these cells at the lipid–water interface.

Various authors have indicated a direct effect of cetirizine on Eos,[2–5] PMN[9,10] and Plt.[11] This study demonstrates that cetirizine can influence membrane fluidity, which plays an important role in modulating cell functions.[28] It is thus plausible that the observed changes in membrane fluidity and heterogeneity induced by cetirizine can alter membrane associated activities of these cells. The fact that Eos, PMN and Plt treated with cetirizine blocked the effect of PAF on membrane fluidity of these cells may be on the basis of the previous observations of a reduction of membrane receptor mediated cell activities triggered by PAF or other agents such as N-formyl peptide or IgE.[5,7,9,11]

This study demonstrates that cetirizine, at a concentration of 1 μg/ml, can influence plasma membrane fluidity of Eos, PMN and Plt. Cetirizine decreases membrane fluidity and heterogeneity at the lipid–water interface. The observed changes can be attributed to a possible interaction of cetirizine with cell membrane components (lipids of proteins) or the cell cytoskeleton that can influence the membrane. The possibility of an interaction of cetirizine with a specific membrane receptor (W. Konig, personal communication) or with nonspecific receptors cannot be excluded. These findings may partially explain the effect of cetirizine on cell functions. Further studies are needed to explain the mechanisms of cetirizine–cell interaction.

### References

1. Campoli-Richards DM, Buckley MMT, Fitton A. Cetirizine: a review of its pharmacological properties and clinical potential in allergic rhinitis, pollen-induced asthma, and chronic urticaria. *Drugs* 1990; **40**: 762–781.
2. Leprevost, C, Capron M, De Vos C, Tomassini M, Capron A. Inhibition of eosinophil chemotaxis by a new anti-allergic compound (cetirizine). *Int Arch Allergy Appl Immunol* 1988; **87**: 9–13.
3. Shemi R, Walsh GM, Hartnell A, *et al.* Modulation of human eosinophil chemotaxis and adhesion by anti-allergic drugs *in vitro*. *Pediatr Allergy Immunol* 1993; **4** (suppl): 13–18.
4. Fadel R, Herpin-Richard N, Rihoux J-P, Henocq E. Inhibitory effect of cetirizine 2HCl on eosinophil migration *in vitro*. *Clin Allergy* 1987; **17**: 373–379.
5. Michel L, De Vos C, Rihoux J-P, Burtin C, Benveniste J, Dubertret L. Inhibitory effect of oral cetirizine on *in vivo* antigen-induced histamine and PAF-acether release and eosinophil recruitment in human skin. *J Allergy Clin Immunol* 1988; **82**: 101–109.
6. Charlesworth EN, Kagey-Sobotka A, Norman PS, Lichtenstein LM. Effect of cetirizine on mast cell-mediator release and cellular traffic during the cutaneous late-phase reaction. *J Allergy Clin Immunol* 1989; **83**: 905–912.
7. Fadel R, David B, Herpin-Richard N, Borgnon A, Rassemont R, Rihoux J-P. *In vivo* effects of cetirizine on cutaneous reactivity and eosinophil migration induced by platelet-activating factor (PAF-acether) in man. *J Allergy Clin Immunol* 1990; **86**: 314–320.
8. Redier H, Chanez P, De Vos C, *et al.* Inhibitory effect of cetirizine on the bronchial eosinophil recruitment induced by allergen inhalation challenge in allergic patients with asthma. *J Allergy Clin Immunol* 1992; **90**: 215–224.
9. Walsh GM, Moqbel R. Hartnell A, Kay AB. Effects of cetirizine on human eosinophil and neutrophil activation *in vitro*. *Int Arch Allergy Appl Immunol* 1991; **95**: 158–162.
10. Kyan-Aung U, Hallsworth M, Haskard D, De Vos C, Lee TH. The effects of cetirizine on the adhesion of human eosoniphils and neutrophils to cultured human umbilical vein endothelial cells. *J Allergy Clin Immunol* 1992; **90**: 270–272.
11. De Vos C, Joseph M, Leprevost C, *et al.* Inhibition of human eosinophil chemotaxis and of the IgE dependent stimulation of human blood platelets by cetirizine. *Int Arch Allergy Appl Immunol* 1989; **88**: 212–219.
12. Coyle AJ, Lefort J, Vargaftig BB. The effect of cetirizine on antigen-dependent leucopenia in the guinea-pig. *Br J Pharmacol* 1991; **103**: 1520–1524.
13. Shinitzky M. Membrane fluidity and cellular functions. In: Shinitzky M, ed. *Physiology of Membrane Fluidity, Vol 1*. Boca Raton, FL: CRC Press, 1984; 1–51.
14. Lenaz G. The role of lipids in the structure and function of membranes. In: Roodyn DB, ed. *Subcellular Biochemistry, Vol 6*. New York: Plenum Press, 1979; 233–343.
15. Kantar A, Wilkins G, Swoboda B, *et al.* Alterations in the respiratory burst of polymorphonuclear leukocytes from diabetic children. *Acta Paediatr Scand* 1990; **79**: 535–541.
16. Koenderman L, Kok PTM, Hamelink ML, Verhoeven AJ, Bruijnzeel PLB. An improved method for the isolation of eosinophilic granulocytes from peripheral blood of normal individuals. *J Leukocyte Biol* 1988; **44**: 79–86.
17. Kubina M, Lanza F, Cazenave J-P, Laustriat G, Kuhry J-G. Parallel investigation of exocytosis kinetics and membrane fluidity changes in human platelets with the fluorescent probe, trimethylammonio-diphenylhexatriene. *Biochem Biophys Acta* 1987; **901**: 138–146.
18. Kantar A, Giorgi PL, Curatola G, Fiorini R. Interaction between PAF and human platelet membranes: a fluorescence study. *Mediators of Inflammation* 1992; **1**: 127–131.
19. Kantar A, Giorgi PL, Fiorini R. Effect of PAF on human lymphocyte membranes: a fluorescence study. *Agents Actions* 1993; **38**: C115–C117.
20. Kantar A, Giorgi PL, Curatola G, Fiorini R. Alterations in erythrocyte membrane fluidity in children with trisomy 21: a fluorescence study. *Biol Cell* 1992; **75**: 135–138.
21. Kantar A, Giorgi PL, Curatola G, Fiorini R. Effect of PAF on erythrocyte membrane heterogeneity: a fluorescence study. *Agents Action* 1991; **32**: 347–350.
22. Fiorini R, Curatola G. Membrane heterogeneity studied by fluorescence lifetime distributions of DPH and TMA-DPH. *Applied Fluorescence Technology* 1991; **III**: 33–38.
23. Fiorini R, Valentino M, Wang S, Glaser M, Gratton E. Fluorescence lifetime distributions of 1,6-diphenyl-1,3,5-hexatriene in phospholipid vesicles. *Biochemistry* 1987; **26**: 3864–3870.
24. Yeagle P. *The Membranes of Cells*. San Diego, CA: Academic Press, 1987.
25. Spector AA, Yorek MA. Membrane lipid composition and cellular function. *J Lipid Res* 1985; **26**: 1015–1035.
26. Van Der Meer BW. Biomembranes structure and dynamics viewed by fluorescence. In: Hildreson HJ, ed. *Fluorescence Studies on Biological Membranes, Vol 13*. New York: Plenum Press, 1988; 1–53.
27. Lenaz G, Fato R, Barraca A, Solaini G, Parenti-Castelli G, Rabbi A. Dynamics of biological membranes. *Ann Ist Super Sanita* 1988; **24**: 9–21.
28. Kinnunen PKJ. On the principles of functional ordering in biological membranes. *Chem Phys Lipids* 1991; **57**: 375–399.
29. Van Der Meer W. Physical aspects of membrane fluidity. In: Shinitzky M, ed. *Biology of Membrane Fluidity, Vol 1*. Boca Raton, FL: CRC Press, 1984; 53–71.
30. Lentz BR. Membrane 'fluidity' as detected by diphenylhexatriene probes. *Chem Phys Lipids* 1989; **50**: 171–190.
31. Kuhry, Fonteneau P, Duportil G, Maechling C, Laustriat G. TMA–DPH: a suitable fluorescence polarization probe for specific plasma membrane fluidity studies in intact cells. *Cell Biophys* 1983; **5**: 129–140.
32. Collins JM, Grogan WM. Comparison of steady-state anisotropy of the plasma membrane of living cells with different probes. *Biochim Biophys Acta* 1991; **1067**: 171–176.
33. Prendergast FG, Haugland RP, Callahan PJ. 1-[4-(Trimethylamino)phenyl]-6-phenylhexa-1,3,5-triene: synthesis, fluorescence properties, and use as fluorescence probe of lipid bilayers. *Biochemistry* 1981; **20**: 7333–7338.
34. Van Blitterswijk WJ, Van Hoeven RP, Van Der Meer BW. Lipid structural order parameters (reciprocal of fluidity) in biomembranes derived from steady-state fluorescence polarization measurements. *Biochim Biophys Acta* 1981; **644**: 323–332.
35. Pottel H, Van Der Meer W, Herreman W. Correlation between the order parameters and the steady state fluorescence anisotropy of 1,6-diphenyl-1,3,5-hexatriene and an evaluation of membrane fluidity. *Biochim Biophys Acta* 1983; **730**: 181–186.
36. Canonica GW, Pesce G, Ruffoni S, *et al.* Cetirizine does not influence the immune response. *Annals of Allergy* 1992; **68**: 251–254.
37. Kantar A, Oggiano N, Giorgi PL, Fiorini R. Interaction of PAF with the plasma membrane of eosinophils obtained from allergic asthmatic children. *Mediators of Inflammation* 1993; **3**: 92–93.
38. Fiorini R, Curatola, G, Bertoli E, Giorgi PL, Kantar A. Changes of fluorescence anisotropy in plasma membrane of human polymorphonuclear leukocytes during the respiratory burst phenomenon. *FEBS Lett.* 1990; **273**: 122–126.
39. Zannoni C, Arcioni A, Cavatorta P. Fluorescence depolarization in liquid crystals and membrane bilayers. *Chem Phys Lipids* 1983; **32**: 179–250.
40. Fiorini R, Valentino M, Glaser M, Gratton E, Curatola G. Fluorescence lifetime distribution of 1,6-diphenyl-1,3,5-hexatriene reveals the effect of cholesterol on the microheterogeneity of the erythrocyte membrane. *Biochim Biophys Acta* 1988; **939**: 485–492.
41. Parasassi T, Conti F, Glaser M, Gratton E. Detection of phospholipid phase separation: a multifrequency phase fluorimetry study of 1,6-diphenyl-1,3,5-hexatriene fluorescence. *J Biol Chem* 1984; **259**: 14011–14017.
42. Kalusner RD, Kleinfeld AM, Hoover RL, Karnovsky MJ. Lipid domains in membranes: evidence derived from structural perturbations induced by free fatty acids and lifetime heterogeneity analysis. *J Biol Chem* 1980; **255**: 1286–1295.

**Received 28 February 1994; accepted in revised form 29 March 1994**
